# Crude Extract from *Ziziphus Jujuba* Fruits, a Weapon against Pediatric Infectious Disease

**Published:** 2013-01-22

**Authors:** F Daneshmand, H Zare-Zardini, B Tolueinia, Z Hasani, T Ghanbari

**Affiliations:** 1 Department of Biology, Payame Noor University, Po Box 19395-3697, Tehran, I. R. Iran.; 2 Young Researchers and Elite Club, Yazd Branch, Islamic Azad University, Yazd, Iran.; 3 Department of Pediatric Hematology, Oncology and Genetics Research Center, Shahid Sadoughi University of Medical Sciences and Health Services, Yazd, Iran.; 4 University of Applied Science and Technology of Sistan and Baluchestan, minushargh Branch, Zahedan, Iran.; 5 Department of chemistry, Faculty of Sciences, Ferdowsi University of Mashhad, Mashhad, Iran.; 6 Iranian Academic Center for Education, Culture and Research (ACECR), Mazandaran Branch, Sari, Iran.

**Keywords:** Fungi, Pediatrics, *Ziziphus*

## Abstract

**Background:**

Pediatric infectious disease is one of the main problems in cancerous children that treat by chemotherapy drugs. Thus, study in this regard is necessary. The aim of this study was to evaluate antimicrobial properties of ethanolic extract of *Ziziphus Jujuba *fruits against different infectious pathogens.

**Materials and Methods:**

This study is descriptive. In vitro antimicrobial activity of extract was assessed on gram negative and gram positive bacteria as well as fungi. The antimicrobial activity was tested by Radial Diffusion Assay (RDA) and Minimal Inhibitory Concentration (MIC) methods.

**Results:**

The results showed a wide antimicrobial activity of the extract against the microbes studied. *Escherichia coli* was the most susceptible to the extracts among tested microorganisms for which the MIC was 0.65±0.22 mg/ml. Amongst the bacterial strains investigated, *Staphylococcus aureus *was the most resistant strain with MIC of 2.26±0.68 mg/ml. The ethanolic extract also showed antimicrobial activity on the fungi studied as no growth was observed in 2.35±0.38 and 2.86±0.7 mg/ml concentration for *Candida albicans* and *Aspergillus fumigatus, *respectively. The results of qualitive and quantitative test are well indicative of the extract effective activity against the microbes mentioned.

**Conclusion:**

Confirming the potential antimicrobial activities of crude extract of *Ziziphus Jujuba* fruits, this study suggested that ethanolic extracts of this plant is appropriate candidate for treatment of microbial infections, especially pediatric infectious diseases.

## Introduction

Immune deficiencies following cancer treatment with different chemotherapy drugs such as doxorubicin lead to development of infectious diseases, especially in children (1); hence, search and discovery over antimicrobial compounds has been an active branch of medical sciences. Use of traditional antibiotics such as penicillin causes development of resistant microbial strains. This status is more than important for children’s infections. With increasing antibiotic-resistant microbial strains, the discovery of new antimicrobial compounds has been of double significance (2). Various sources of poisonous secretion of animals such as snake, scorpion, spider, amphibians, insects and etc have been discovered with diverse effects (3-6). The plants are also of special importance. Herbal plants, in particular, have a special place in traditional medicine. Antimicrobial herbal compounds are one of the valuable medical resources, and in line with the spread of pediatric infectious diseases, identification of more of these extracts and compounds will be useful in treating patients (7, 8). Plant-derived antimicrobial compounds have numerous therapeutic effects; not only they are useful for treatment of infectious diseases, but also have less side effects than most of antimicrobial compounds (9-11). Isolation of effective antimicrobial components has been made from various plants, showing a significant impact on a variety of pathogenic bacteria and fungi. Historically, plants have provided sources of antimicrobial compounds to fight infection (12, 13). It is estimated that 250000 to 500000 plant species exist on Earth, some of which are widely used in medicine (14). In recent years, the attention has been attracted to research on medicinal plants. Various activity-related aspects of plant compounds have been investigated against various bacteria, fungi, viruses and protozoa. Hexan extract of the stem bark of *Amona glabra* (15), alkaloid extract from dried seeds of *Semecarpus anacardium (*16*)*, alcoholic extract of the stem bark of *Clausena anisata (*16*)*, aqueous extracts of *Azadirachta indica* leaves (17) and oil extract from the foliage of *Santolina chamaecyparissus* and *Aegle marmelos* (18) have shown antimicrobial activity against various bacteria and fungi. *Zizyphus jujuba* has medicinal properties; it is used as antidote, diuretic, laxative and expectorant. Its dried fruits are used as sedatives, anticancer, antipyretic, analgesic, appetizer, anti-hemorrhage agents and as the tonic as well (19-21). So far, there is no study on plant extract against pediatric infection. Thus, the present study has been conducted to evaluate antimicrobial activity of raw extract of *Ziziphus Jujuba* fruits.

## Materials and Methods

This study is descriptive. Sample extraction was carried out through Soxhlet extractor. For this purpose, 15 g powder of *Zizyphus jujuba* fruits was filled in the thimble and extracted with ethanol solvent in Soxhlet extractor for 48 hours (22). Then the solution was filtered by filter paper. The extract was concentrated to one-tenth of the original volume using the vacuum distillation. To investigate the antimicrobial activity, two methods, including Radial Diffusion Assay and macro broth dilution, were used. The extract was dissolved in distilled water. The antimicrobial effects of the extract were investigated using Radial Diffusion Assay (RDA) as described below.

Two species of gram-positive bacteria (*Bacillus subtilis *and *Staphylococcus aureus*) and two species of gram-negative bacteria (*Escherichia coli *and *Pseudomonas aeruginosa*) were used for primary assays. An aliquot of bacteria with a titer of 4 × 10^6^ CFU was mixed with 10 ml of medium containing 0.03% TSB and 1% agarose and was poured into a plate. Holes were then created in the medium using a punch, 10 µl of the plant extract was loaded into the wells and the plates were incubated for 3 h at 37 °C. After the three-hour incubation, the secondary medium, enriched with 6% TSB and 1% agarose was poured into the plate, and the plates were incubated at 37 ºC for 18 hours. The plate was then stained for 24 hours using a solution containing 37% formaldehyde, 15 ml; methanol, 27 ml; water, 63 ml; and Coomassie brilliant blue R-250, 2 mg. The plates were destained for approximately 10 min with an aqueous solution of 10% acetic acid and 2% dimethylsulfoxide (23). 

Antifungal activity of the plant extract was tested on *Candida albicans and Aspergillus fumigatus*. One ml of fungal suspension was inoculated in 20 ml of Potato dextrose agar and was poured into the germ culture plates. The holes were then created by the punch in the medium and were filled by 10 µl of the plant extract. The plates were incubated for 7 days at 30°C to 35°C and the results were recorded during this period (24). 

To determine the MIC, stock serial dilutions of 1 to 35 mg/ml of the extract were prepared and 20μl of the extract stocks were added to a solution containing 10^6^ CFU/ml of bacteria, which was then poured into a plate. The microplate was incubated at 37 °C for 18 hours. After this time, the absorbance of each well was read at 630 nm using an Enzyme-Linked Immunosorbent Assay (ELISA) reader, and the results were compared to the control samples. The MIC was defined as the extract concentration at which the absorbance of the treated bacterial sample is half of that of the untreated well of bacteria. Escherichia coli, *Pseudomonas* *aeruginosa, Staphylococcus aureus *and* Bacillus subtilis*, were used for MIC determination. Experiments were carried out in triplicate.

To determine the fungi-associated MIC, 180 microliters of Sabouraud Dextrose Agar culture medium along with 10 microliters of fungal suspension (10^6^ CFU/ml) and 10 microliters of serial concentration of the plant extract were poured in microplates which were then incubated at 37 °C for 24 hours. The MIC was similarly defined as minimum concentration at which no growth was observed. *Candida albicans* and *Aspergillus fumigatus* were used for MIC determination. Experiments were carried out in triplicate (24, 25).

## Results

The results showed that plant extract obtained from *Zizyphus jujuba* fruits has inhibitory effect on the four bacteria studied. This plant extract has also antimicrobial activity against the fungi investigated in this study.

**Figure1:**
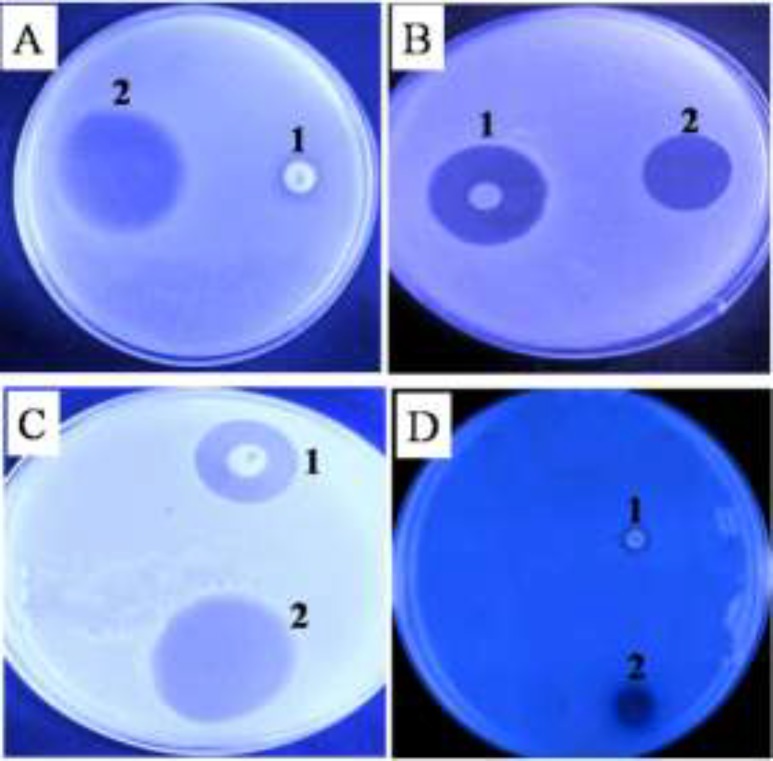
Antimicrobial effects of ethanolic extract against gram-positive and gram-negative bacteria. 1, vancomycin; 2,ethanolic extract of Zizyphus jujuba fruits. A, Escherichia coli; B, Bacillus subtilis; C, Pseudomonas aeruginosa; D, Staphylococcus aureus

**Figure 2:**
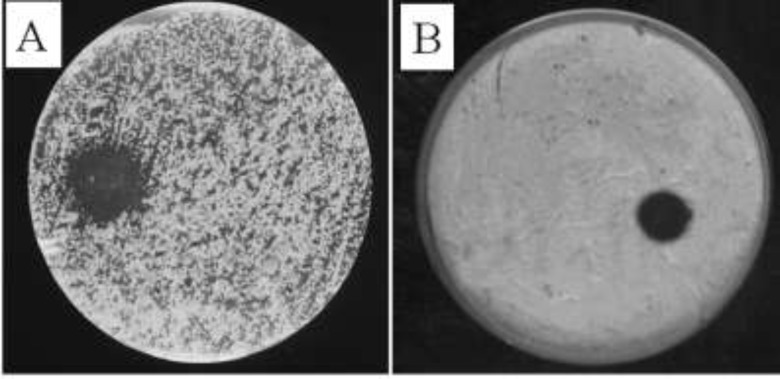
Antimicrobial effects of ethanolic extract against fungi. A, Candida albicans; B, Aspergillus fumigatus

The qualitative assessment results on antibacterial activity of the plant extract are shown in Figure1. The results indicate the effective antibacterial activity of the plant extract against the bacterial strains studied. As clear from qualitative results, *Staphylococcus aureus *and *Escherichia coli *have respectively displayed the most and the least sensitivity to the extract. As shown in Figure 1, extract-related diameter of the inhibition zone is more than that of vancomycin antibiotic, demonstrating more effective antibacterial activity of the extract. The plant extract also revealed antifungal activity, so as it led to significant fungal destruction in the area around the injected site at concentration of 1mg/ml (Figure 2). 

As mentioned before, MIC was determined for quantifying antimicrobial activity of *Zizyphus jujuba* fruits extract on the microbes investigated, and the results are presented in Table I. As shown at Table I, the results of the quantitative test confirmed the results of qualitative test, as *Staphylococcus aureus *and *Escherichia coli *were introduced as the most and the least sensitive bacterial strains in quantitative test. As shown in Table I, the plant extract also has potent antimicrobial activity against two fungal strains. 

**Table I:** The MIC values of ethanolic extract.

microbe	MIC(mg/ml)
*Bacillus cereus*	1.5±0.45
*Staphylococcus aureus*	2.26±0.68
*Escherichia coli*	0.65±0.22
*Pseudomonas aeruginosa*	0.76±0.32
*Candida albicans*	2.35±0.38
*Aspergillus fumigatus*	2.86±0.7

## Discussion

Plant extracts are a complex combination of different chemical elements with different values. Due to significant alteration in these compounds, biological effects of the extracts are varied. Antimicrobial properties of some herbal extracts has been identified (26-28); regarding these properties and other biological impacts, plant extracts have been of great attention as an appropriate substitute for antibiotics for potential therapeutic targets (29, 30) and/or health-cosmetics products and food industry (31). 

*Zizyphus jujuba* is a pharmaceutical plant with proven therapeutic potentials; investigation on antimicrobial effects of the plant essential oils has shown a highly effective antibacterial activity (19-21). The results of the present study is also demonstrating antimicrobial effects of *Zizyphus jujuba* fruit extract, which is in line with other researches confirming medicinal effects of this plant (32, 33). Besides the antibacterial effects, findings also revealed antifungal activity of the extract, so as it eliminated the *Candida albicans* and *Aspergillus fumigatus* completely effectively at low concentrations. The results exhibited that in addition to antimicrobial activity of jujube oils as well as therapeutic potentials of other parts of the plant such as leaves, jujube fruit can also be of biological effectiveness, particularly in terms of antibacterial and antifungal activity (33, 34). 

The present study, in which macro broth dilution and disk diffusion were applied, demonstrated more effective antibacterial property of jujube fruit extract against the gram negative and gram positive bacteria strains. 

Findings also showed effective antimicrobial activity of the extract on the four bacteria studied in comparison with Vancomycin antibiotic. In this study, it has been observed that jujube extract has less antimicrobial effect on gram positive bacteria. This data are similar with other articles that examine similar extract (35, 36). Fungi also are sensitive to plant extract. In general, herbal products contribute to cytoplasm granulation (37), cytoplasmic membranes rupture (38) and deactivation or inhibition of intracellular and intercellular enzyme activity, cell walls disintegration and the destruction of electron transport system (39); these cellular events can independently or simultaneously reach to a maximum level while preventing the mycelial growth (3). Mechanism of antifungal activity of jujube fruit extract may be owing to any of the reasons mentioned. Crude Extracts from *Ziziphus Jujuba* Fruits has effective antimicrobial impact against gram negative and gram positive bacteria as well as fungi, so as it revealed more effective antimicrobial activity compared to common antibiotic like Vancomycin. The results of the present study have clearly showed acceptable antimicrobial effect of this plant extract against fungi in addition to gram positive and gram negative bacteria. Thus, this plant extract may be suitable for treatment of infectious diseases, particularly for Pediatric infection.
